# Comparison of Continuous Versus Interrupted Chest Compressions during CPR in a Rural Community

**Published:** 2018-11-29

**Authors:** Gregory M. Thomas, James T. Prescott

**Affiliations:** University of Kansas School of Medicine, Department of Family and Community Medicine, South Central Medical Education Network Site, McPherson, KS

**Keywords:** resuscitation, cardiopulmonary resuscitation, resuscitation orders, emergency medical service, rural hospital

## Abstract

**Introduction:**

Cardiopulmonary resuscitation (CPR) in patients with out-of-hospital cardiac arrest (OHCA) have interruption of manual chest compressions for airway management and breathing when performed by medical personnel trained by Advanced Cardiac Life Support (ACLS) standards. This interruption likely reduces blood flow and possibly survival. Traditional CPR (30:2 compressions to ventilations) was compared with continuous chest compressions, CCC (also termed Cardiocerebral Resuscitation, CCR) in a rural community.

**Methods:**

A retrospective cohort analysis of three years of traditional CPR (June 2008 – May 2011) for OHCA was compared to three years of using CCC protocols (June 2011 – May 2014). Primary outcomes were survival at one and six months.

**Results:**

There were 58 0HCA patients in the six year study period (June 2008 – May 2014). Forty (69%) received CPR and 18 (31%) received CCC. Two (5%) survived at least one month with CPR and eight (44%) survived at least one month with CCC (p = 0.0007). After six months, 0/40 (0%) who received CPR had survived and 6/18 (33%) who received CCC survived (p = 0.0018). For the patient found in ventricular fibrillation or tachycardia (a shockable rhythm), 0/13 (0.0%) survived one month after CPR and 7/9 (78%) survived with CCC (p < 0.01). After six months 0/13 (0.0%) survived with CPR and 6/9 (67%) survived with CCC (p < 0.05).

**Conclusions:**

For patients in a rural environment with OHCA, CCC had a more favorable outcome than traditional CPR. For the patient found in ventricular fibrillation or ventricular tachycardia, there was a profound survival benefit of CCC over CPR.

## INTRODUCTION

Heart disease is the leading cause of death in the United States.^1^ Out-of-hospital cardiac arrest (OHCA) is often a precursor of cardiac death.^2^–^4^ To date, the most common care of patients with OHCA by health professionals is initiation of cardiopulmonary resuscitation (CPR) and advanced cardiac life support (ACLS). Since 2008, the recommendation for nonmedically trained bystanders has been chest compression only CPR.^5^–^7^ According to the American Heart Association (AHA) in 2015, the United States had greater than 350,000 OHCA victims, 46.1% received bystander CPR but only 12% survived.^8^

Currently, physicians, nurses, nurse practitioners, physician assistants, and emergency medical personnel are taught 30:2 compressions to respirations ratio as initial resuscitation efforts.^8^ Even with immediate treatment by bystanders or first responders, survival remains dismal. OHCA survival has remained low for several decades.^3^ Non-traumatic cardiac arrest with any rhythm treated by emergency medical services (EMS) had a survival to the hospital of 7.3%.^1^ Survival of those found in ventricular fibrillation with bystander CPR was 31.4%.^1^ Clearly, any intervention that provides hope to prolong ventricular fibrillation and perfusion is worth repeated evaluation.

McPherson County, Kansas is a rural community of approximately 29,000 people, located 60 miles north of Wichita, the closest large urban center. McPherson Hospital serves the local community with 41 licensed beds, an emergency department staffed with board certified emergency medicine physicians or family physicians. McPherson emergency medical services provide paramedic led emergency services to the county as well as back up to smaller volunteer services throughout the area. It is staffed with paramedics trained in basic and advanced cardiac life support.

**Figure 1:**
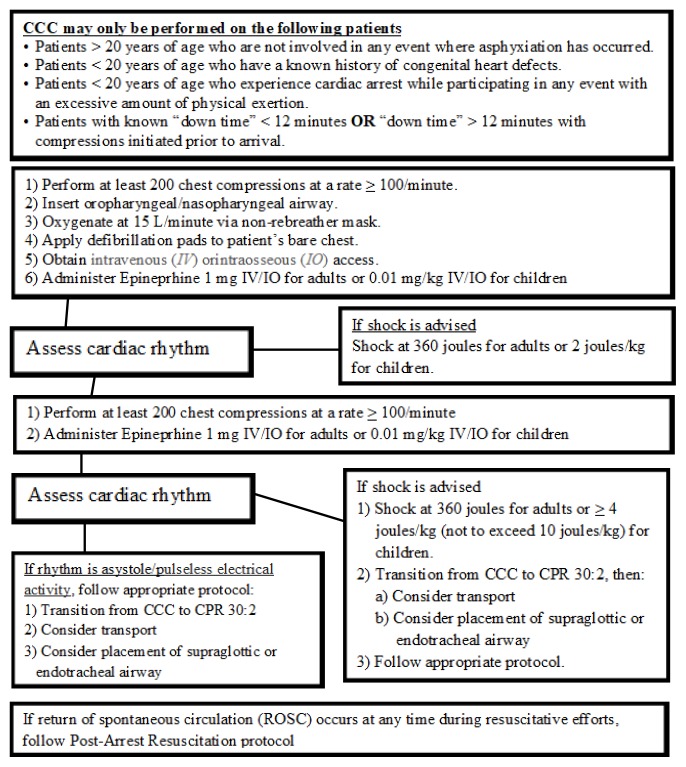
McPherson Emergency Medical Service protocol for continuous chest compressions (CCC).

CCC (as defined in Figure 1) provides chest compressions only during the first several cycles of resuscitation, with timely defibrillation and pharmacotherapy when available.^9^ CCC is at least equivalent if not superior to standard CPR in laboratory studies as well as cohort studies.^9^–^24^

By early 2011, the McPherson medical staff and EMS personnel became committed to transitioning to CCC for OHCA. This was based on a review of the data^9^–^24^ and personal contact with Dr. Gordon A. Ewy, an early proponent of CCC.

This project evaluated traditional American Heart Association CPR performed over three years to three years of using CCC following OHCA in a rural environment. The primary end points were out-of-hospital survival at one and six months.

## METHODS

A retrospective cohort analysis compared traditional CPR used from June 2008 to May 2011 to the CCC protocol used from June 2011 to May 2014. Data were collected from the cardiac arrest database maintained by the EMS service as well as hospital records. Date of arrest, gender, race, type of resuscitation performed, time of call, time of initiation of resuscitation efforts, time of first epinephrine dose, time of the return of spontaneous circulation (ROCS), time from initial 911 call to initiating CPR or CCC, time from initiating CPR or CCC to return a spontaneous circulation and final patient outcomes were collected.

Prior to 2011, traditional ACLS protocols for CPR were used by trained medical personnel. Beginning January 2011 initial education was conducted with medical staff, EMS crews, first responders, emergency dispatch operators, and interested members of the community. This education consisted of presentations covering the rationale of CCC, education regarding the technique, and finally practical application of the new skill set.

After six months of education, beginning in June 2011, the CCC protocol (Figure 1) was initiated as standing orders for county wide EMS crews (responders to the OHCA event). Starting in December 2011, using proprietary 911 phone instructions, emergency dispatchers instructed callers (bystanders to the OHCA event) to start chest compressions with no breaths (ACLS standard for bystanders since 2008)^5^–^7^ on patients who were deemed to be experiencing OHCA until EMS arrived on scene. Chest compressions quality and timing was dictated by AHA recommendations. Initial airway management included insertion of an oral or nasopharyngeal airway, administration at 15 L per minute of oxygen via a non-rebreather mask to provide passive oxygenation. Only after three rounds of 200 chest compressions (at a rate of 100 compressions per minute) were advanced airway techniques and positive pressure ventilation considered by EMS providers (Figure 1). Post resuscitation care was provided at the discretion of the emergency department physician upon arrival to the hospital.

### Statistical analysis

Differences in categorical data, such as mortality at one and six months were calculated using the Fisher’s Exact test. Differences in mean values, such as age and time to events, were calculated using the Student t test. P value less than 0.05 was considered statistically significant.

This project was approved by the Institutional Review Board of the University of Kansas School of Medicine.

## RESULTS

There were 58 non-traumatic OHCA patients in the six-year study period (June 2008 – May 2014). Study demographics and pertinent population differences are shown in Table 1; study results are shown in Tables 2 and 3. From June 2008 until May 2011, there were 40 patients with OHCA who received traditional CPR. From June 2011 until May 2014, there were 18 who received CCC. The mean age was 68 years. The only statistical significant difference was earlier administration of epinephrine in the CPR group.

Bystander CPR was reported if it was initiated within ten minutes of the 911 call. There was a trend toward the CCC time period having a higher percentage of bystander CPR (50% vs. 32.5%) but it was not statically significant.

Two (5%) survived at least one month with CPR and eight (44%) survived at least one month with CCC (p = 0.0001). After six months, 0/40 (0%) who received CPR had survived and 6/18 (33%) who received CCC survived (p = 0.002).

Thirteen patients in the CPR group were in ventricular fibrillation or tachycardia. Seven survived but lived less than 30 days, usually only one or two days. None lived past 30 days. Of the nine CCC patients with ventricular fibrillation or tachycardia, all lived 30 days, seven lived one to six months, and six lived over six months. Those CCC patients who survived to leave the hospital were all confirmed to be neurologically intact. Others have documented a similar result.^25^ Neurologic status was confirmed by interview with patients or family concerning level of function in 2017, if alive, compared to prior to OHCA event.

## DISCUSSION

The implementation of CCC in our rural community has been a welcome change at all levels. Paramedics who had practiced over 20 years and never had a long term survivor with a field save, immediately experienced field saves with good outcomes. One of the most striking results was that those who were in a shockable rhythm (ventricular fibrillation or ventricular tachycardia) and received CCC, 100% regained a pulse in the field and 67% survived long term. CPR resulted in fewer field saves and no one survived long term.

The expectation is that the patient who is found in ventricular fibrillation or ventricular tachycardia will survive. As with the rest of the country, the participation rate for bystander CPR was low and most likely contributed to increased morbidity and mortality. This improved some with the implementation of the 911 phone advice protocols six months into the CCC study period, but this was not statistically significant. The EMS staff and the greater medical community were accepting of the new CCC protocol as presented after a six month education process. This process is reproducible. The outcome will need confirmation with larger numbers.

Our study had some weaknesses. Bystander 911 protocols were initiated six months after CCC protocols. Bystander CPR, therefore, may have been more effective, adding to the survival rate.^25^–^26^ Time of administration of epinephrine was earlier on average in CPR. The data were retrospective and sample size small even over six years since it was collected in a rural county. However, this confirms work done by Garza et al.^21^ Of note, one of the investigators was a survivor of a cardiac arrest event in which CCC was utilized by bystander and EMS personnel during the study period.

The 2017 ILCOR^27^ summary statement notes knowledge gaps in three areas for OHCA:

1. What is the effect of delayed ventilation versus high quality CPR?
2. Which elements of the bundled care (compressions, ventilations, delayed defibrillation) are most important?
3. How effective is passive oxygen insufflation?

This study provides a limited retrospective look at these issues in the rural environment.

It has long been recognized that keeping or finding patients in a shockable rhythm (ventricular fibrillation or ventricular tachycardia) is the key to good outcome.^1^ It has been theorized that within the first ten minutes of a cardiac event with loss of circulation, the red cells carry an adequate amount of oxygen.^7^, ^10^, ^12^ It also has been theorized that stopping chest compressions, even briefly, to give a breath causes loss of perfusion and therefore oxygenation.^7^,^10^,^12^–^16^ If this is the case, CCC has the potential to extend the period of successful defibrillation electrically to up to ten minutes.^7^,^10^,^12^–^16^ These are valuable minutes that could make survival possible.^7^,^10^,^12^–^16^ While the numbers are small, the experience in McPherson has been dramatic and statistically significant. A small but important change in how we approached the patient with cardiac arrest has yielded an important outcome that should be reproducible in any rural community. Our statistical OHCA survival in a small rural environment compares favorably to the standard that is published the best urban centers.^28^–^29^ Going forward, our community has made it a focus to make sure that we have defibrillators available to fire rescue, police and sheriff departments, churches, sporting arenas and any area that has large numbers of individuals in one place. Donations and foundation support has been raised to help with this.

While it took us six months to implement this protocol, with help from leaders in this area such as Dr. Ewy, the protocol is simple. It took acceptance from medical and emergency personnel. It should be noted that most of the data in the literature pertained to out of hospital arrests. No conclusions should be reached for the hospitalized patient based on these data.

For the patients in ventricular defibrillation or tachycardia there was dramatic survival benefit, lending credence to the possibility of CCC prolonging the window for successful defibrillation.

## Figures and Tables

**Table 1:** Demographics and pertinent differences between subjects receiving CPR versus CCC.

Demographics	CPR	CCC	Total	p value
Average years	69.6	64.5	68	> 0.05
Female patients	9 of 40	1 of 18	10/58 (17.2%)	0.114
Time of epinephrine administration (n) if given	5.85 (36)	9.00 (14)	7.48 min. (40)	.007*
Presented in ventricular fibrillation and tachycardia	13 of 40 (32.5%)	9 of 18 (50%)	22/58 (37%)	0.25
Average time to CPR or CCC by trained EMS personnel	6.275 min. (40)	6.07 min. (18)	6.21 min. (58)	0.765
CPR or CCC performed by a bystander	13 of 40 (32.5%)	9 of 18 (50%)	22/58 (37%)	0.249
Average time to bystander CPR or CCC	2.25 min. (n = 12)	1.0 min. (n = 9)	1.71 min.	0.236

* Results are statistically significant.

**Table 2:** Study results for subjects receiving CPR versus CCC.

Outcome	CPR	CCC	Total	p value
Time of the return of spontaneous circulation in minutes (n)	17.23 (17)	17.20 (10)	17.21 (27)	0.89
Survived 1 – 30 days	13 of 40 (32.5%)	11 of 18 (61%)	24/58 (43%)	0.0495*
Survived 1 – 6 months	2 of 40 (5%)	8 of 18 (44%)	10/58 (17%)	0.001*
Survived over 6 months	0 of 40 (0%)	6 of 18 (33%)	6/58 (10%)	0.002*
CPR or CCC performed by trained EMS personnel	13 of 24 (54%)	9 of 13 (69%)	22/37 (59%)	0.373
Left hospital alive after bystander response	1 of 13 (7.7%)	4 of 9 (44%)	5/22 (23%)	0.116

* Results are statistically significant.

**Table 3:** Patients with ventricular fibrillation/tachycardia with ROSC.

Outcome	CPR	CCC	Total	p value
Survived 1 – 30 days	7/13 (54%)	9/9 (100%)	16/22 (73%)	0.074
Survived 1 – 6 months	0/13 (0%)	7/9 (78%)	7/22 (31%)	< 0.01*
Survived over 6 months	0/13 (0%)	6/9 (67%)	6/22 (27%)	< 0.05*

* Results are statistically significant.
